# Guidelines for the management of asthma in adults and adolescents: Position statement of the South African Thoracic Society – 2021 update

**DOI:** 10.7196/AJTCCM.2021.v27i4.189

**Published:** 2021-12-31

**Authors:** U G Lalloo, I S Kalla, S Abdool-Gaffar, K Dheda, C F N Koegelenberg, M Greenblatt, C Feldman, M L Wong, R N van Zyl-Smit, on behalf of the Asthma Working Group of the South African Thoracic Society

**Affiliations:** 1 Life Mount Edgecombe Hospital, Durban, South Africa; 2 Division of Pulmonology, Department of Internal Medicine, Faculty of Health Sciences, University of the Witwatersrand, Johannesburg, South Africa; 3 Kingsway Hospital, Amanzimtoti, South Africa; 4 Centre for Lung Infection and Immunity,Division of Pulmonology, Department of Medicine and UCT Lung Institute and South African Medical Research Council/UCT Centre for the Study of Antimicrobial Resistance, University of Cape Town, South Africa; 5 Faculty of Infectious and Tropical Diseases, Department of Immunology and Infection, London School of Hygiene and Tropical Medicine,United Kingdom; and Division of Pulmonology, Department of Medicine, Stellenbosch University, Cape Town, South Africa; 6 Milpark Hospital, Johannesburg, South Africa

**Keywords:** asthma, guidelines, management

## Abstract

Asthma prevalence is increasing worldwide, and surveys indicate that most patients in developed and developing countries, including South Africa, do not receive optimal care and are therefore not well controlled. Standard management guidelines adapted to in-country realities are important to support optimal care. The South African Thoracic Society (SATS) first published a guideline for the management of chronic persistent asthma in 1992, which has subsequently been revised several times.

The main aim of the present document was to revise and update SATS’ statement on the suggested management of chronic asthma, based on the need to promote optimal care and control of asthma, together with the incorporation of new concepts and drug developments. This revised document reinforces optimal care and incorporates the following primary objectives to achieve the recent advances in asthma care:

- continued emphasis on the use of inhaled corticosteroids (ICS) as the foundation of asthma treatment
- to reduce the reliance on short-acting beta-2 agonist (SABA) monotherapy for asthma symptoms
- to incorporate the evidence and strategy for the use of the combination of an ICS and formoterol for acute symptom relief (instead of a SABA)
- to incorporate the evidence and strategy for the use of as-needed ICS-long-acting beta agonists (LABA) for patients with infrequent symptoms or ‘mild’ asthma
- to incorporate the evidence and strategy for the use of a long-acting muscarinic antagonist (LAMA) in combination with ICS-LABA; and
- to incorporate the evidence and strategy for the use of and management with a biologic therapy in severe asthma.

## Background

Asthma prevalence is increasing worldwide, and surveys indicate that most patients in developed and developing countries, including South Africa (SA), do not receive optimal care and are therefore not well controlled.^[1]^ Standard management guidelines adapted to in-country realities are important to support optimal care.^[1]^ The South African Thoracic Society (SATS) first published a guideline for the management of chronic persistent asthma in 1992, which has subsequently been revised several times.^[2–4]^

## Objectives

The main aim of the present document was to revise and update SATS’ statement on the suggested management of chronic asthma, based on the need to promote optimal care and control of asthma, together with the incorporation of new concepts and recent developments in pharmacotherapy. The management guidelines for acute asthma exacerbations have not changed substantially and are presented elsewhere.^[5]^ The present revision stresses optimal care and incorporates the following objectives to achieve the recent advances in asthma care:

- continued emphasis on the use of an inhaled corticosteroid (ICS) as the foundation of asthma treatment and control.
- to minimise the need for, and/or inappropriate reliance on, short-acting beta-2 agonist (SABA) monotherapy for acute asthma symptoms.
- to incorporate the evidence for, and strategy for the use of, the combination of an ICS and formoterol for acute symptom relief (instead of a SABA).
- to incorporate the evidence and strategy for the use of as-needed ICS-long-acting beta agonist (LABA) for patients with infrequent symptoms or ‘mild’ asthma.
- to incorporate the evidence and strategy for the use of a long-acting muscarinic antagonist (LAMA) in combination with ICS-LABA in patients with symptoms unresponsive to ICS-LABA alone.
- to incorporate the evidence and strategy for the use of, and management with, a biologic therapy in severe asthma.
- to clarify the need for clinical and laboratory phenotyping of difficult-to-treat asthma.
- to clarify the role of bronchial thermoplasty (BT) for severe and difficult-to-treat asthma; and
- to incorporate recommendations for COVID-19 disease and prevention in patients with asthma.

## Key points

1. SA has one of the highest reported asthma mortality rates, despite availability of ICS in all sectors of the healthcare system.
2. The cornerstone of asthma treatment remains ICS.
3. Patients with so-called ‘mild’ asthma are at risk for acute exacerbations and death, and should have an ICS included in their management strategy.
4. Early diagnosis and control of asthma will reduce morbidity and mortality, and most people with asthma can lead a normal life with optimal control.
5. The combination of an ICS with a LABA may control asthma with a lower maintenance dose of the ICS, and is recommended for most asthma patients.
6. Patients with infrequent symptoms (less than 4 - 5 days a week) can be safely treated with an as-needed combination of ICS-formoterol.
7. Where possible, the combination ICS and formoterol, rather than salbutamol alone, should be recommended for acute symptom relief, particularly in patients not on long-term ICS-LABA.
8. The complacency with frequent salbutamol usage needs to be addressed by education and increased and regular use of an ICS.
9. Biologic therapies (monoclonal antibodies directed towards IgE, interleukin-5/5-receptor (IL-5/5r), interleukin-4 receptor (IL-4r), etc.) are increasingly becoming available and need to be prudently prescribed after specialist review and clinical phenotyping.
10. Slow-release theophylline should be considered only as an add-on fourth-line controller.
11. Long-term use of oral corticosteroids is strongly discouraged owing to their severe side-effect profile.
12. Bronchial thermoplasty may be considered for severe or difficult-to-control patients with asthma on optimal medical treatment at a specialist referral centre.
13. Home nebulisers are discouraged for the control of asthma and are not a substitute for optimal control with recommended treatment strategies.
14. Inhaler technique and adherence must be addressed at every consultation before making any change in drug therapy. Poor technique is one of the most common causes of poor asthma control.
15. Lung function should be measured annually in patients with asthma to objectively measure lung function impairment. At each clinic visit, a simple and validated scoring method such as the Asthma Control Test (ACT) should be used to determine current asthma control.
16. Where possible, patient preference should be considered in the selection of inhaler device (dry powder or pressurised metered dose inhaler (pMDI)). Adequate instruction in inhaler technique is essential and should be checked at every visit.
17. Well-controlled asthma does not appear to be a risk factor for severe acute respiratory syndrome coronavirus 2 (SARS-CoV-2) infection and coronavirus disease 2019 (COVID-19). Poor asthma control requiring the use of oral corticosteroids may increase the risk for COVID-19 disease.

## Position statement development

Based on new evidence and clinical trials of asthma treatment, a working group of clinicians and researchers based in SA revised and updated the previous asthma guideline.^[4]^ Changes were based on the levels of evidence outlined in Table 1 and in accordance with the Global Initiative for Asthma (GINA) 2021 strategy document.^[1]^

The field of asthma is changing rapidly, with many new drugs becoming available. The availability of medications and local approvals should be reviewed prior to prescription. The recommendations are guided by the 2021 GINA strategy document and reflect best practice, with alternatives when availability is restricted by registration/cost/ funder restrictions/patient preference.

**Table 1:** Categories of evidence for management strategies in asthma

**Evidence level**	**Source of evidence**
**A**	Randomised controlled trials (RCTs), and/or systematic reviews, observational evidence. Rich body of data
**B**	Few RCTs and systematic reviews. Limited body of data.
**C**	Non-randomised trials and observational studies
**D**	Panel consensus judgement

RCT = randomised controlled trials

## Definition of asthma

The GINA definition states that asthma is ‘a heterogeneous disease, usually characterised by chronic airway inflammation’. It is defined by ‘the history of respiratory symptoms such as wheeze, shortness of breath, chest tightness and cough that may vary over time and in intensity, together with variable airflow limitation which may later become permanent.’^[1]^

Several asthma phenotypes have been described and are recognised by their clinical and pathophysiological features. These are important when considering the option of prescribing a biologic agent.

Common phenotypes seen in clinical practice include:

- ***Allergic (extrinsic) asthma:*** This is the most common phenotype. Childhood onset is common, with a personal or family history of atopic disorder such as allergic rhinitis, allergic conjunctivitis, eczema, asthma, or food and drug allergy. It is characterised by eosinophilic airway inflammation and generally has a good response to inhaled corticosteroids.
- ***Non-allergic (intrinsic) asthma:*** No evidence or history of allergy. May have neutrophilic inflammation with less response to an ICS.
- ***Late onset/adult-onset asthma:*** Presents with asthma for the first time in adulthood. Usually non-allergic and may require a higher dose of ICS.
- ***Asthma with ‘fixed airflow limitation’:*** Usually long-standing asthma with poor control and failure to normalise lung function owing to airway remodelling despite intensive treatment.

Other sub-phenotypes (which may fit into the above but with special features):

- ***Exercise-induced asthma (EIA):*** Exercise-induced symptoms may be the only manifestation of asthma in young patients with allergic asthma. Managed with usual asthma therapies.
- ***Cough variant asthma:*** Cough is the major manifestation of asthma and more common in children. Managed with usual asthma therapies.
- ***Asthma with obesity:*** Increasingly more common, in line with trends in the global obesity epidemic, with high symptom frequency and less responsive to an ICS.
- ***Work-related asthma:*** Asthma caused (occupational asthma) or aggravated (work-aggravated asthma) by exposure to agents in the workplace. A detailed occupational history is essential to suspect occupational asthma. Once diagnosed, removal from exposure is an essential step. While some patients’ asthma may improve following removal from exposure, the asthma may persist despite removal from exposure. Treatment of asthma is according to standard guidelines. Patients with occupational asthma are entitled to compensation under SA labour law and should be referred to centres of expertise in occupational health.^[6]^
- ***Catamenial asthma:*** One-fifth of women with asthma may have premenstrual worsening of symptoms.
- ***Aspirin-exacerbated respiratory disease:*** Aspirin may cause exacerbations in those with aspirin sensitivity previously known as aspirin-induced asthma. Patients with suspected aspirin sensitivity should be referred to a specialist.

## Steps in the diagnosis and management of asthma

**1. Symptoms and signs of asthma**

The characteristic symptoms of asthma are wheeze, shortness of breath, tightness of the chest and cough that vary over time and in intensity.

Patterns of symptoms that suggest asthma are:

- variability: day and night, day to day, seasonal
- precipitation by a range of factors including environmental allergens (house dust mite, grass pollens, animal dander, occupational exposures), non-specific irritants (smoke, dust and fumes), cold weather and exercise
- symptomatic response to a bronchodilator and an ICS.

An expiratory wheeze is a cardinal sign of asthma but may be absent at the time of consultation. Airway constriction is variable, does not always result in detectable signs, and may not be present at the time of the consultation. A personal history of other atopic disorders such as allergic rhinitis, allergic conjunctivitis and eczema is supportive evidence that the respiratory symptoms are due to asthma. A positive family history of asthma and other atopic disorders are helpful supportive evidence. However, not all people with asthma are atopic, particularly late-onset asthma.

A history of current or past cigarette smoke exposure is important as cigarette smoking is the primary cause of chronic obstructive pulmonary disease (COPD), which needs to be differentiated from asthma. Furthermore, smoking affects asthma control and response to an ICS. Patients with asthma may have features of COPD and vice versa, which may be referred to as asthma-COPD overlap (ACO).^[7]^

**2. Lung function testing**

Spirometric lung function tests, including measurement of peak expiratory flow, are key to the diagnosis, assessment of severity, management and monitoring of asthma. Normal lung function does not exclude a diagnosis, especially in well-controlled asthmatics, and particular note should be paid to patients with significant signs and symptoms but normal spirometry.

Reduction in forced expiratory volume in 1 second (FEV_1_) and peak expiratory flow (PEF) are the hallmark of asthma but may be seen in other diseases. A ratio of FEV_1_ to forced vital capacity (FVC) below 70% or the lower limit of normal (LLN) is characteristic of obstructive airways disease. Significant bronchodilator responsiveness (reversibility) with normalisation of the airway obstruction is the major physiological characteristic of asthma. The standardised criteria for bronchodilator responsiveness are an increase in FEV_1_ of >12% and 200 mL, 10 - 15 minutes following the inhalation of 200 - 400 µg of salbutamol, or a 20% improvement in PEF from baseline. The degree of reduction of FEV_1_ is generally related to severity of the airway obstruction. Asthma improvement is usually mirrored by an improvement in FEV_1_ and PEF.

Not all asthma patients will exhibit bronchodilator responsiveness at presentation; this could be due to recent bronchodilator treatment and patients should be advised to withhold their SABA for 6 hours, twice-daily LABA for 24 hours, and once-daily LABA for 36 hours prior to testing. The test may need to be repeated at different visits to detect variability in lung function. Alternatively, demonstrating a diurnal variation in PEF may be of value. An average of more than 10% variation over 2 weeks is indicative of asthma. This can also be used to identify environmental (including occupational) causes of asthma symptoms by monitoring PEF 2 - 4 times each day for at least 2 weeks. To measure diurnal variation, PEF is measured first thing in the morning and in the evening. There are several methods of calculating diurnal PEF variability. A useful method is the difference between the maximum and the minimum value for the day, expressed as a percentage of the mean daily PEF value, and averaged over 2 weeks.

Asthma can also be confirmed by demonstrating increased hyperresponsiveness to bronchoconstrictor stimuli. This is usually only of value in subjects with near-normal spirometry. A methacholine/ histamine challenge test should only be undertaken in a specialist lung function laboratory. Alternatively, airway hyperresponsiveness may be demonstrated using an exercise test for exercise-induced bronchoconstriction. A fall of 20% in PEF (or decrease >10% and 200 mL in FEV_1_ ) from baseline within 30 minutes after a 6-minute run and having achieved >80% of the predicted heart rate for age, is supportive of a diagnosis of asthma.^[8]^ Allergy testing may be supportive evidence of asthma but is not specific and not all people with asthma are atopic. Measurement of exhaled nitric oxide (FeNO) is not routinely recommended for the diagnosis of asthma in adults.^[1]^ Lung function testing options are summarised in Table 2.

Patients who are on anti-inflammatory treatment (ICS) for asthma may not demonstrate features of asthma with any of the lung function tests. If it is essential to confirm a diagnosis of asthma, it may be necessary to reduce the dose of treatment gradually and repeat the tests after 4 weeks of a dose reduction. SABA for symptom relief should be used in the interim. Use of a SABA must be avoided for at least 6 hours prior to repeat testing.

**Table 2:** Lung function tests to determine reversibility of airway obstruction or bronchial hyperresponsiveness to confirm a diagnosis of asthma*

**Test**	**Method**	**Diagnostic criteria**	**Comment**
**Spirometry FEV_1_**	Administer 200 -400 μg salbutamol. Measure FEV_1_ before and 10 - 15 min. after administration	12% and 200 mL improvement in FEV_1_	Standard test for all asthma if spirometry available. COPD may show similar changes in early stages
**PEF (with PEF meter or spirometer)**	Administer 200 - 400 μg salbutamol. Measure PEF before and 10 - 20 min. after administration	20% improvement in PEF	Readily available test in primary care
**Exercise testing**	Aerobic exercise for ~6 min. Measure PEF or FEV_1_ within 30 min. post exercise	10% and 200 mL decrease in FEV_1_	-
**Methacholine/histamine challenge**	Measure FEV_1_ before and after inhalation of increasing doses of methacholine or histamine	Lowest provocative dose causing a 20% drop in FEV_1_ (PD20)	To be undertaken in specialist laboratories with resuscitation facilities

FEV_1_ = forced expiratory volume in 1 second

PEF = peak expiratory flow

COPD = chronic obstructive pulmonary disease

* Bronchodilators must be stopped before conducting a lung function test to diagnose asthma: 6 hours for SABA, 24 hours for twice-daily LABA, and 36 hours for once-daily LABA or LAMA

**3. Laboratory testing**

Routine laboratory tests are generally not required to make a diagnosis of asthma. A full blood count with a differential white cell count may demonstrate eosinophilia. Serum immunoglobulin E (IgE) may be raised, and serum allergen testing may be indicative of allergic sensitisation. In severe and difficult-to-control asthma, phenotyping is important, and the above tests should be performed. Measurement of FeNO is a surrogate marker of allergic inflammation but may be raised in many other conditions, and is not recommended in routine practice.^[9]^

**4. Differential diagnosis**

Many conditions may present with symptoms similar to asthma. These must be excluded with a careful history and examination and particularly if the criteria for a diagnosis of asthma are not fulfilled. Table 3 summarises the conditions that frequently mimic asthma and asthma symptoms, using an anatomical approach.

**Table 3:** Differential diagnosis of airway obstruction

**Location of airway obstruction**	**Differential diagnosis to consider**
**Diffuse airway** • presents with diffuse wheeze	Asthma COPD Acute and chronic bronchitis Bronchiolitis Left ventricular failure/pulmonary congestion
**Large airway (tracheal obstruction) and vocal cords** • trachea and left and right main bronchus • may present with stridor	Extrinsic compression (thyroid, lymph nodes) Lesions in the lumen or wall (stenosis, stricture, tumour) Cartilage (tracheomalacia, relapsing polychondritis) Vocal cord dysfunction
**Bronchial obstruction** • may present with focal wheezes	Extrinsic compression (lymph nodes) Lesions arising from the wall (tumour. stenosis, endobronchial TB, sarcoidosis) Endobronchial lesion such as a foreign body

COPD = chronic obstructive pulmonary disease

TB = tuberculosis

Cough as an isolated symptom is common in clinical practice and is also a common symptom of asthma. Upper respiratory tract infections, usually caused by viruses may cause a cough without wheeze, and usually remits within 6 weeks. Chronic rhinitis and sinusitis with a postnasal drip may also present with a chronic cough and may be the first manifestation of atopy with asthma developing later.

Upper airway obstruction is a serious condition and requires urgent investigation and management. It may present with stridor that is often mistaken for a wheeze unless one auscultates carefully and times the ‘wheeze’ which occurs during inspiration. A localised wheeze must prompt investigations for focal lung disease, including endobronchial obstruction. Diffuse wheezes are present with acute and chronic bronchitis, COPD, left ventricular failure, and bronchiolitis.

It is important to distinguish asthma from COPD because of the different treatment approach and prognosis. Many people with asthma who smoke develop COPD, and patients with COPD may develop asthma. This overlap has received much attention as the coexistence of both asthma and COPD frequently makes the control of symptoms more difficult. There are no formal diagnostic criteria but patients with features of both conditions could be labelled as ACO if for no other reason than to identify the potential challenge in treatment and to default to an asthma-led treatment approach.

## Assessment of asthma severity or control

The evaluation of asthma ‘severity’ has recently undergone a paradigm shift with the recognition that so-called ‘mild’ asthma may be associated with significant mortality, even though symptoms are infrequent or ‘mild’.^[1]^ Additionally, in patients who have a diagnosis of asthma and are already on asthma treatment, the diagnosis of severity is based on intensity of treatment that is required to control symptoms (and exacerbations) rather than the frequency of symptoms, which simply reflect ‘control’. For those potentially with truly severe asthma characterised by ongoing symptoms despite at least medium-dose ICS-LABA combination therapy, a specific workup should be conducted to confirm the need for high-dose corticosteroids or biologic therapy. (Further details are provided in the section below on special considerations in asthma management.)

The principles of asthma therapy are to achieve asthma control, which may be defined as: no night-time waking or limitation in daily activities, and symptoms occurring no more than twice a week or requirement for SABA reliever usage less than twice a week.^[1]^ The ACT may also be used to evaluate level of control with a score >20/25 considered to be indicative of well-controlled asthma. ^[10]^

## Treatment of asthma

### Initial choice of therapy

Patients presenting with infrequent symptoms and without significant lung function impairment, smoking history or recent hospitalisation should be commenced on as-needed low-dose ICS-formoterol (Evidence B). If this option is not available, a low-dose inhaled corticosteroid should be taken whenever a SABA is used via separate inhalers (Evidence B).^[1]^ (Table 4)

**Table 4:** Initiation of asthma treatment

**Presentation**	**Choice of initiation medication**
**Infrequent symptoms <2 times a month**	As-needed low-dose ICS-formoterol *or* as-needed SABA and low-dose ICS whenever the SABA used
**More frequent symptoms (2 - 4 times a month)**	As-needed low-dose ICS-formoterol *or* regular low-dose ICS and as-needed SABA
**Recurrent (almost daily) symptoms, night waking and/or risk of exacerbations**	Low-dose ICS-LABA and as-needed SABA *or* low-dose ICS-formoterol as regular maintenance and use as reliever therapy

ICS = inhaled corticosteroid

SABA = short-acting beta agonist

LABA = long-acting beta agonist

If patients present with a new diagnosis of asthma with significantly impaired lung function, with symptoms occurring frequently and/or night wakening due to asthma, regular low-dose ICS-LABA with a SABA as reliever, or as-needed low-dose ICS-formoterol should be initiated to gain control of the symptoms and prevent further lung function impairment (Evidence A). In patients who present with an acute exacerbation, a short course of oral corticosteroids may be required to gain control in parallel with the initiation of regular therapy. Where possible, ICS-formoterol may be used as the reliever as well as controller – or a SABA can be used as a reliever in conjunction with the ICS (Evidence A).^[1]^ (Table 4)

Patients should be reviewed within 2 - 3 months to ensure that they have responded to the medication. Inhaler techniques and comorbid conditions – particularly allergic rhinitis and gastro-oesophageal reflux – should be reviewed and treated optimally.

### Choice of treatment options for long term

***Selecting maintenance treatment***

**1. Set goals for asthma treatment**

The emphasis of modern asthma treatment is to achieve control and minimise future risk. With optimal treatment, patients should be able to live a normal life with a normal life expectancy. Basic goals are to:

- achieve and maintain control of symptoms.
- maintain normal activity levels, including exercise, and sleep uninterrupted by asthma symptoms.
- maintain near-normal pulmonary function.
- prevent asthma exacerbations.
- avoid adverse effects from asthma medications.
- prevent asthma morbidity and mortality.

**2. Patient education and environmental control**

Patient education about asthma is a critical step in the management. There must be agreement with patient and care provider about the goals of asthma treatment. Better long-term maintenance of asthma control and outcomes have been achieved where the patient and clinician have jointly set the goals for asthma treatment. The SA National Asthma Education Programme (NAEP) and GINA have published information leaflets for asthma patients.^[11,12]^

Wherever possible, identified asthma triggers must be avoided. These include active and passive cigarette smoke exposure, animal dander and pollen. Air pollution, workplace asthma triggers and dust exposure may all trigger asthma symptoms. Routine testing for allergies is not recommended all people with asthma. It should be considered in individuals with difficult-to-control symptoms, specific exposures potentially needing intervention (work related) or suspected dairy or food product/preservatives exacerbating asthma. Maintaining a clean, well-ventilated indoor environment is advisable but expenditure on extensive changes to carpeting, bedding etc. should only be done after consultation with a specialist. Aspirin and non-steroidal anti-inflammatory agents should be avoided in patients known to have aspirin-sensitive asthma. Non-cardio-selective beta blockers (oral) should be avoided and only instituted in exceptional circumstances and under specialist supervision. Even ophthalmic beta blockers may worsen asthma control and should be used with caution. If cardio-selective beta blockers are required for ischaemic heart disease, the decision to prescribe should be discussed, given the potential benefits and risks.^[13]^

Inhaler technique is critical to ensure optimal delivery of inhaled medication, and significant time must be devoted to ensuring that patients understand and know how to use their inhaler treatment. Inhaler technique must be reviewed at every visit and, if persistent errors cannot be rectified, an alternative inhaler device should be considered. Spacer devices improve the delivery of inhaled medications where inhaler-breathing coordination is problematic. Spacers should ideally be used by all patients using a pressurised metered dose inhaler (pMDI)-type device, particularly for ICS-containing medications to ensure full benefit from the treatment.

Peak flow monitoring and recording of symptoms in an asthma diary are increasingly possible using a variety of electronic Apps in addition to a traditional paper-based diary. These diaries and reminders support adherence and provide the attending clinician with more data to inform management decisions. A personalised asthma plan is an important part of asthma management detailing when to adjust medication and when to seek medical help. These may be of great value to patients with particularly difficult-to-control symptoms.

**3. Pharmacotherapy**

Asthma therapy can broadly be classified into three groups:

**3.1. Controller medication.** These medications contain an inhaled corticosteroid and may be paired with a long-acting bronchodilator. The goal with these medications is to reduce airway inflammation and to improve symptoms and lung function, thus reducing the need for a reliever.^[14–16]^ Leukotriene receptor antagonists and theophylline are also considered controller therapies but are less effective and used as third- or fourth-line agents.
**3.2 Reliever medication.** These medications have traditionally been short-acting bronchodilators with rapid onset of action that provide acute relief of symptoms. Recent evidence now allows the use of a fast-onset long-acting bronchodilator – such as formoterol – in combination with an ICS,^[15,16]^ and this is the preferred strategy as put forward by GINA, but may be limited by cost and availability.^[1]^
**3.3 Adjunctive controller medication.** These medications may be added to the ICS-LABA regimen in patients in whom symptoms remain uncontrolled despite medium-dose ICS-LABA. They generally are reserved for more severe asthma patients not controlled by at least medium-dose ICS-LABA. They include LAMAs, azithromycin, oral corticosteroids, and biologic therapies (monoclonal antibodies to IgE, IL-5/5r, IL-4r etc.)

Inhaled medications for asthma are available as pMDIs, breath-actuated metered-dose inhalers, dry powder inhalers (DPIs), soft-mist inhalers and nebulisers. Patient preference and ability to use a device should, where possible, be accommodated, given cost and formulary constraints. It requires training and practice to effectively co-ordinate activation of a pMDI with inhalation. Patients having difficulty using a pMDI are recommended to use a large-volume (500 mL) spacer to improve drug delivery to the lungs and reduce local side-effects. A pMDI plus spacer, or a DPI, is as effective as a nebuliser for delivering a SABA (Evidence A).^[17]^

## Available therapies

A large variety of controllers are available globally and, depending on locally approved formularies, may be available in SA. Table 5 contains a breakdown of controllers shown to be effective in large clinical trials. (Not all medications listed in Table 5 for use in asthma have been approved in SA at the time of publication of this guideline; regulatory approval must be confirmed prior to prescribing.)

**Table 5:** Controller medication formulations available for asthma*

**ICS**	**LABA**	**Adjunctive controller**
Budesonide Fluticasone propionate Ciclesonide Beclomethasone dipropionate (BDP)	**12 hour action:** Salmeterol Formoterol	Leukotriene receptor antagonists • Montelukast • Zafirlukast
Mometasone		Slow-release theophylline

**ICS-LABA combinations**	**ICS-LABA-LAMA combinations**	**LAMA**

**12-hour action:** Fluticasone propionate-salmeterol Budesonide-formoterol Beclomethasone-formoterol Mometasone furoate-formoterol **24-hour action:** Fluticasone furoate-vilanterol Mometasone furoate-indacaterol	Mometasone furoate-indacaterol-glycopyrronium Fluticasone furoate-vilanterol-umeclidinium Beclomethasone dipropionate-formoterol-glycopyrronium Budesonide-formoterol-glycopyrronium	Tiotropium

ICS = inhaled corticosteroid

LABA = long-acting beta agonist

LAMA = long-acting muscarinic antagonist

*These medications were developed for use in asthma but may not be available or registered for use in asthma at the time of publication of these guidelines. Regulatory approval must be confirmed before prescribing.

### Reliever therapies for asthma

Relievers are short-acting bronchodilators that are used for acute symptom relief. SABAs are the most commonly prescribed reliever medications and include salbutamol, fenoterol and terbutaline.

SABAs provide relief from acute symptoms of asthma and are usually prescribed as 200 μg salbutamol as needed (Evidence A). Patients with well-controlled asthma require use of a reliever less than twice a week. Overreliance/abuse of SABAs has been associated with significant mortality and should strongly be discouraged.^[18–20]^ Current evidence suggests that using an anti-inflammatory-reliever combination (ICS-formoterol) in mild asthma is superior to a SABA alone and should be considered as first choice in patients with mild asthma taking intermittent medication only.^[15–16]^ In more severe disease, the ICS-formoterol combination can continue to be used as reliever as well as maintenance, if on the same maintenance inhaler (so-called MART – maintenance and reliever therapy).^[21]^

In patients using non-ICS-formoterol maintenance therapy, such as fluticasone-salmeterol, there are no data to support safety or efficacy of using ICS-formoterol as the reliever, and a SABA should be used as the reliever in this situation.^[1]^

Short-acting anti-muscarinic (SAMA) drugs such as ipratropium bromide work by inhibiting vagally mediated bronchoconstriction and are not the preferred reliever in asthma. They may be used in patients, particularly the elderly, who cannot tolerate SABA side-effects, or patients who are intolerant of SABA.^[1]^

### Anti-inflammatory agents

**Inhaled corticosteroids (ICSs)**

Anti-inflammatory treatment with an ICS is recommended for all patients with chronic asthma.^[1]^ Their long-term use in optimal doses decreases exacerbations and mortality.^[18]^ Systemic absorption of an ICS arises from oropharyngeal absorption and to a lesser extent from drug deposited in the lungs. This may be reduced with the use of a spacer device combined with rinsing of the mouth after inhalation (Evidence A). The former increases the fraction delivered to the lung (Evidence A). Both measures reduce the incidence of local side-effects such as dysphonia and oropharyngeal candidiasis. DPIs cannot be used with a spacer. Ciclesonide may be considered for patients with local ICS side-effects as it is a pro-drug and activated only in the lung.

ICS therapy can be administered once or twice daily as single medications or in combination with a LABA and LAMA. Doses of ICS are classified as ‘low, medium or high’. These are not potency equivalence, but broad groupings based on clinical effect from trial evidence.^[1]^ When in combination with a LABA or LAMA, the dosing may further vary, and manufacturer package leaflets should be consulted to clarify dosing. Low-dose (total daily dose) ICS include: budesonide 200 - 400 μg, fluticasone propionate 100 - 250 μg, or ciclesonide 80 - 160 μg. Medium daily doses are: budesonide 400 - 800 μg, fluticasone propionate 250 - 500 μg, or ciclesonide 160 - 320 μg. High-dose ICS: budesonide >800 μg, fluticasone propionate >500 μg or ciclesonide >320 μg.^[1]^ There are individual and combination inhalers with varying doses of ICS. A comprehensive table detailing the individual and combination corticosteroid containing inhalers can be found in the GINA guidelines: Box 3 - 6 ‘Low, medium and high daily metered doses of inhaled corticosteroids (alone or with LABA)’.^[1]^ This table provides a comparison of ‘low, medium and high’ ICS doses and should be consulted when clarity is required on a particular inhaled ICS, device or combination.

At higher doses, the dose-response curve of ICSs is relatively flat, with increased risk of systemic side-effects such as growth retardation, skin bruising, cataracts, diabetes mellitus, dyslipidaemia, Cushing syndrome and osteoporosis. When used in combination with a LABA and LAMA, a lower dose of ICS is needed for the same clinical outcome, thus reducing total exposure to corticosteroids. The majority of asthma patients should achieve adequate symptom control with low- or medium-dose regimens of ICS. Those requiring long-term higher doses of ICS to control symptoms should be referred to a specialist in asthma care for review. Strategies to minimise osteoporosis such as regular exercise, calcium supplementation and hormonal replacement in postmenopausal women, should be considered.

Nebulised corticosteroids are expensive, require high-pressure nebulisers (not ultrasonic) for optimal delivery, and are not recommended for routine use in chronic asthma. Long-term oral corticosteroids should only be considered in patients with severe asthma refractory to treatment after all other treatment options have been exploited, and preferably with referral to a specialist centre for evaluation. International guidelines^[1]^ recommend using a biologic therapy rather than long-term oral corticosteroids.

If asthma control is achieved with the combination of low-dose budesonide with formoterol twice daily, then the option of MART should be considered. With this strategy, the same inhaler is used for daily maintenance therapy as well as reliever therapy, thus avoiding two separate inhalers. This method is only possible with formoterol-containing combinations owing to formoterol’s fast onset of action and safety profile. Salmeterol is not suitable owing to its slower onset of bronchodilation and a safety concern when administered in other than the recommended dose.

### Sustained action bronchodilators

**Long-acting beta-2 agonists (LABAs)**

Salmeterol and formoterol are LABAs that are administered twice daily because of their longer duration of action but should never be used without co-administration of an ICS. Formoterol, a LABA with a rapid onset of action similar to a SABA, has a linear dose response curve and, in combination in a single inhaler, can be used both as a reliever and as maintenance therapy.^[21]^ Formoterol doses up to 96 µg/day have been used without major adverse events. Ultra-LABAs, with a duration of action over 24 hours, are available in combination with an inhaled corticosteroid as a once-daily option.^[22,23]^ Salmeterol is not suitable for acute relief of asthma symptoms because it has a delayed onset of action and is limited by the ceiling dose of 50 µg twice daily. Side-effects of LABA drugs include palpitations, tremor, nausea and nervousness.

### Long-acting muscarinic antagonists (LAMAs)

LAMAs are recommended as monotherapy for patients with moderate COPD.^[24]^ In asthma, when added to maintenance treatment with ICS alone or ICS/LABA, they improve lung function and may reduce the risk of exacerbations. Their effect on asthma symptoms is less consistent.^[25–27]^ They should be considered in patients who are uncontrolled on medium- to high-dose ICS-LABA.^[1]^

There are currently no data to guide the clinician’s decision on whether to add a LAMA or ‘step up’ to a higher dose of ICS in a patient uncontrolled on medium ICS-LABA. Prescription of a LAMA should be considered in patients who are uncontrolled on high-dose ICS-LABA.^[1]^

### Adjunctive controller therapies

***Leukotriene receptor antagonists (LTRAs)***

LTRAs inhibit the leukotriene pathway of asthma inflammation but have weak overall anti-inflammatory effects compared with low-dose ICSs.^[28]^ They have been shown to improve asthma control and exert their effect within days of commencing treatment. They may be of benefit in aspirin-exacerbated and catamenial asthma. LTRAs are not recommended as monotherapy in ‘mild’ asthma. They should instead be considered as add-on treatment in patients on an ICS regimen. If no benefit is evident after 4 weeks, the LTRA should be withdrawn because not all patients respond. Patients should be counselled about neuropsychiatric side-effects.^[29]^

***Slow-release (SR) theophylline***

The role of oral SR theophylline in the control of asthma is limited and should only be considered in patients with severe or difficult-to-treat asthma not responsive to inhaled therapies. Formulations of SR theophylline have a 12-hour, and some a 24-hour, duration of action. Their disadvantages include a narrow therapeutic range, drug interactions and frequent side-effects (nausea, vomiting, abdominal pain, gastro-oesophageal reflux, palpitations, insomnia, irritability and seizures).^[30,31]^

***Low-dose oral corticosteroids***

Chronic oral corticosteroids are associated with significant long-term side-effects and should only be considered when all other options have been exhausted. The lowest possible dose should be used and preferably never more than 10mg prednisone equivalent per day.

***Azithromycin***

Add-on azithromycin given thrice weekly has been shown to reduce exacerbations in patients taking high-dose ICS-LABA (Evidence B).^[32,33]^ Significant side-effects include potential cardiac events (QT prolongation), antimicrobial resistance and gastro-intestinal intolerance may occur. Azithromycin 500 mg three times a week should only be initiated after specialist review in patients who are poorly controlled on high-dose ICS-LABA combination therapy.

### Non-pharmacological interventions

In addition to managing the direct airway inflammation and symptoms, several other interventions have been shown to benefit people with asthma and should be considered:

- smoking cessation not only reduces future risk, but also improves response to therapy
- avoidance, where possible, of occupational and environmental pollution
- avoidance of allergic sensitisers – animal dander/pollen, etc.
- vaccination for influenza, pneumococcus and COVID-19
- written asthma action plan to encourage appropriate self-management.

### Biologic therapies

In patients who are uncontrolled on medium- to high-dose ICS combinations, treatment with a targeted biologic therapy is now possible. Asthma phenotypes, referred to as atopic/non-atopic or type-2 high/type-2 low, may respond to specific therapies targeting the underlying asthma pathway. Type-2 high inflammation is characterised predominantly by cytokines such as IL-4, IL-5 and IL-13, which are produced by the adaptive immune system in response to allergens.^[34]^ Some of the common biomarkers used to identify type-2 inflammation include blood and sputum eosinophil counts as well as fractional excretion of nitric oxide (FeNO) levels.^[35]^

Prior to considering the use of a biologic therapy, the patient must be reviewed by a specialist. Adherence, inhaler technique, the presence and treatment of comorbid diseases, and elimination of extrinsic triggers (smoking, allergies) must be considered prior to prescribing these agents. In addition simple phenotyping should be performed to confirm the presence of type-2 inflammation, which serves both as an indication for currently approved biologics and predicts a favourable response. Type-2 status is suggested by elevated blood eosinophils, elevated serum IgE, evidence of atopy (confirmed from history and/ or skin prick or serum specific IgE to common allergens) and/or elevated FeNO.^[36]^ The biologic therapies currently registered in several countries abroad are shown in Table 6.

**Table 6:** Summary of biologic therapies and criteria*

**Biologic target**	**Anti-IgE**	**Anti-IL-5/IL5r**			**Anti-IL-4r**	
**Biologic therapy**	Omalizumab	Mepolizumab	Benralizumab	Reslizumab	Dupilumab	
**Asthma indication**	Severe allergic asthma	Severe eosinophilic asthma			Severe eosinophilic /Type 2 asthma	OCS-dependent severe asthma
**Other indications**	Nasal polyposis, chronic idiopathic urticaria	Hypereosino- philic syndrome, eosinophilic granulomatosis with polyangiitis	NA	NA	Chronic rhinosinusitis with nasal polyposis, atopic dermatitis	

**Eligibility criteria (all criteria marked with a tick must be met)**						

**Exacerbations in previous year**	🗸	🗸	🗸	🗸	🗸	🗸
**Allergic sensitisation to inhaled allergen (SPT or specific-IgE)**	🗸					
**Total serum IgE and body weight within specified range**	🗸					
**Blood eosinophil count above specified threshold**		🗸	🗸	🗸		
**Type 2 biomarkers (e.g. blood eosinophil or FeNO) above specified threshold**					🗸	

SPT = skin prick testing

IgE = immunoglobulin E

FENO = fractional exhaled nitric oxide

OCS = oral corticosteroid

NA = not applicable

*Regulatory approval and approved indications/eligibility criteria must be confirmed prior to prescription

***Biologic therapies available for asthma***

**Anti-IgE (omalizumab)**

Omalizumab is a monoclonal antibody to circulating IgE. It has been available for over 20 years and is used in patients with type-2 high asthma and specifically requires documentation of a raised serum IgE level as it is dosed based on body weight and IgE level.^[37]^ Omalizumab has been extensively used and recent data have shown safety in pregnancy and additional indications such as nasal polyposis.^[38,39]^

**Anti-IL-5 (mepolizumab/reslizumab) anti-IL-5r (benralizumab)**

Mepolizumab and reslizumab target IL-5 and have been shown to reduce exacerbations, but efficacy is restricted to patients with an eosinophil count greater than 150 and 400 cells/µL, respectively in patients on medium- to high-dose ICSs. Benralizumab targets IL-5r and similarly requires patients to have a raised eosinophil count >300 cells/µL treated with high-dose ICS.^[40–42]^

**Anti-IL-4r (dupilumab)**

Dupilumab targets the IL-4 receptor and blocks both IL-4 and IL-13. It reduces exacerbations in patients with and without raised blood eosinophils on a background of medium- or high-dose ICS.^[43,44]^ It has been licensed for use in atopic dermatitis and in patients on medium/ high-dose inhaled or oral corticosteroids.

Several other biological therapies are under development, including anti-TSLP, IL-17, IL-33, etc. but have not been approved widely for clinical usage. Anti-TSLP appears to be of value in those with both TH2-high and TH2-low phenotypes. These newer therapies have a role in the management of poorly controlled asthmatic patients who have frequent exacerbations; however, their cost is currently a significant barrier to their widespread use.

**Bronchial thermoplasty (BT)**

BT refers to the application of radio-frequency-generated heat energy to large and medium-sized airways.^[45]^ It is only recommended in patients with severe uncontrolled asthma who are not responsive to inhaled therapies. Patients who cannot access, or have failed biologic interventions (e.g. anti-IgE, anti-IL-5 and anti-IL-4 immunotherapy), may be considered for BT. It is recommended only to be performed in referral centres with experience in severe asthma and the procedure. Patients should be counselled about the efficacy and long-term safety of BT, uncertainties surrounding BT, and the potential for BT to cause an exacerbation or initial worsening of their symptoms. Recommendation for the use of BT in SA have been published.^[46]^ The lowest possible dose is ideally <10 mg prednisone equivalent per day. Table 7outlines the recommended stepwise approach to asthma therapy with options for treatment choices at each step.

**Table 7:** Stepwise approach to asthma therapy

**Steps 1 and 2: Low-dose ICS therapy**

• As-needed low-dose ICS-formoterol* *Alternatively* • As-needed SABA – and additional ICS taken on each occasion that SABA is used *or* • Regular low-dose ICS with SABA as reliever If the patient remains uncontrolled, review adherence and triggers and step up therapy if indicated.

**Step 3: Medium-dose ICS therapy**

• Low-dose ICS-formoterol as regular maintenance and reliever *Alternatively* • Low-dose ICS-LABA as regular maintenance and SABA reliever *or* • Medium-dose ICS as regular maintenance with SABA reliever If the patient remains uncontrolled, review adherence and triggers and step up therapy if indicated.

**Step 4: Medium- to high-dose ICS therapy with or without additional controllers**

• Medium-dose ICS-formoterol as regular maintenance and low-dose ICS-formoterol as reliever *Alternatively* • Medium-dose ICS-LABA as regular maintenance and SABA reliever • Consider the addition of a LAMA (as single separate component or single inhaler triple combination), LTRA or sustained-release theophylline If the patient remains uncontrolled on step 4 therapy, they should be reviewed by a specialist in asthma care; phenotyping should be performed, and consideration given to additional alternative controllers, biologic therapy, or other interventions^†^

**Step 5: High-dose ICS therapy with or without additional controllers and biologic therapies**

• High-dose ICS-formoterol regular maintenance with low-dose ICS-formoterol as reliever ± separate LAMA *or* • High-dose ICS-LABA with SABA as needed *or* • Medium- or high-dose ICS-LABA-LAMA with SABA as needed^§^ *and* • Consider addition of azithromycin/LTRA/theophylline/low-dose oral corticosteroids • Consider biological therapy if uncontrolled on inhaled therapies: anti-IgE, anti-IL-5/5r, anti-IL-4r, etc. • Consider bronchial thermoplasty^¶^

ICS = inhaled corticosteroid

SABA = short-acting beta agonist

LABA = long-acting beta agonist

ICS = inhaled corticosteroid

LAMA = long-acting muscarinic antagonist

LTRA = leukotriene receptor antagonist

IgE = immunoglobulin E

IL-5/5r; interleukin-5/5 receptor

IL-4r = interleukin-4 receptor

* In ‘mild’ asthma, the preferred option is as-needed ICS-formoterol to ensure patients receive ICS; if not possible, the use of a SABA with co-administration of an ICS is recommended.

† Phenotyping includes evaluation of eosinophils, allergies, IgE levels etc. to support the use of biologic therapies or additional controllers.

§ No data exist for the safety/utility of using an ICS-formoterol as reliever with once-daily therapies or non-formoterol-containing maintenance therapy.

¶ The use of bronchial thermoplasty should be restricted to specialist centres and is detailed in a separate SATS document.^[46]^

## Special considerations in asthma management

### COVID-19

Well-controlled asthma, without significant comorbidities, not requiring high-dose ICS or regular oral corticosteroids, and not having frequent acute exacerbations requiring extra bursts of systemic corticosteroids, is not associated with a higher risk of infection with the severe acute respiratory syndrome coronavirus-2 (SARS-CoV-2) virus or its complications. Individuals with severe asthma or uncontrolled asthma have a greater risk of dying from COVID-19 infection and therefore asthma should be managed appropriately with inhaled corticosteroids. Those receiving biologic therapies should continue treatment. Asthma treatment must not be stopped as asthma exacerbations will worsen COVID-19 disease risks.

People with asthma should continue to respect COVID rules of social distancing, mask wearing and obtain COVID vaccination. Guidance on the use of nebulisers and lung function testing are available on the SATS website (www.pulmonology.co.za).

### Asthma in pregnancy

Asthma control is important and should ideally be optimised prior to conception and maintained throughout pregnancy and during the puerperium. Use of treatment over time has confirmed the safety of most commonly recommended asthma drugs and that the risks of poor asthma control during pregnancy outweigh any potential risk of the usual reliever and controller therapies.^[1,47]^ These include beclomethasone dipropionate, budesonide, SABAs, LABAs, ipratropium bromide, theophylline and oral corticosteroids. They should not be stopped during pregnancy as poor asthma control poses a greater risk to mother and child than any unlikely adverse effect of these treatments. In general, asthma control remains the same in one-third of pregnant asthma patients, worsens in one-third and improves in one-third, and may vary during each of the three trimesters and with subsequent pregnancies. This warrants regular asthma assessment during pregnancy. Optimal asthma control is associated with the best pregnancy outcomes. Of the newer biologic therapies, some safety data exist for omalizumab in pregnancy.^[38]^

### Vocal cord dysfunction

Vocal cord dysfunction may mimic asthma and or worsen asthma symptoms. Patients may have typical asthma symptoms without significant airflow limitation when tested. Visual inspection of the cords may reveal paradoxical movement of the cords. Explanation and counselling are important and symptoms may respond to intensive speech therapy.

### Occupational asthma

Industrialisation is paradoxically associated with increased asthma prevalence owing to the increased production of, and exposure to, respirable irritants and allergens. Exposure to these agents may cause or aggravate asthma and must be considered in adult-onset asthma, difficult-to-control or severe asthma. In principle, the diagnosis of occupational asthma requires the diagnosis of asthma and demonstration of a clear association with workplace exposure to a known or potential airway irritant or allergen and asthma symptoms. If occupational asthma is suspected, the patient should be referred to a pulmonologist or a centre of expertise for investigation. Early removal from exposure can significantly mitigate the development of chronic asthma. Occupational asthma related to a proven causative agent is a scheduled occupational disease under the Occupational Diseases in Mines and Works Amendments Acts of 1994 and 2002 and subject to compensation under the Compensation for Occupational Injuries and Diseases Amendments Acts of 1994 and No. 61 of 1997.

### Exercise-induced bronchoconstriction

Exercise is important for overall health and wellbeing but may trigger symptoms of asthma. However, exercising on days with high pollution or pollen counts should where possible be avoided. Pre-exercise use of a short- or long-acting beta agonist and sufficient warm-up reduces the risk of exercise-induced asthma (Evidence A).^[8]^ If an individual is using ICS-formoterol as their reliever, this can be considered for use pre-exercise rather than a SABA (Evidence B).^[48]^ Advising an athlete to avoid triggers such as training on very hot or cold days and avoiding allergen exposure may reduce the risk of bronchospasm. Clinicians must be cognisant of rules and regulations regarding therapeutic use exemptions and, if asthma symptoms remain uncontrolled, the patient should be referred to a specialist for evaluation.

### Irritant-induced asthma (previously known as reactive airways dysfunction syndrome (RADS) if asthma occurs following a single high-level exposure to an irritant)

RADS is a condition that occurs after exposure to a high concentration of an airway irritant, usually in a confined space and owing to an industrial accident in a non-atopic, non-asthmatic person and presents with acute respiratory distress owing to injury to the airways. It may be fatal or result in severe airway obstruction warranting emergency treatment. It often leads to chronic asthma. Irritant-induced asthma may also occur owing to persistent low-dose exposure to airborne irritants over months to years.^[49]^

### Rhinosinusitis

It is estimated that 80% of asthmatics have concomitant rhinosinusitis, and about 50% of patients with rhinosinusitis have asthma. Anatomically, the upper and lower respiratory tracts are embryonically linked and subject to similar inflammatory processes, and secretions from a postnasal drip enter the lower respiratory tract, causing inflammation. Uncontrolled rhinosinusitis can lead to poor asthma control and vice versa. It is recommended that sinus disease is assessed and treated appropriately, particularly if asthma is not well-controlled or severe.

### Gastro-oesophageal reflux disease (GORD)

GORD is a common cause of heartburn and dry cough and may contribute to poor asthma control. In patients with GORD, treatment with a proton pump inhibitor is recommended (Evidence A). GORD may worsen vocal cord dysfunction which may mimic asthma. Empiric treatment of GORD without symptoms in uncontrolled asthma patients is not recommended as it is has not been shown to improve the asthma control.^[1]^

### Asthma-COPD overlap (ACO)

Asthma and COPD may exist in the same patient. It may be difficult to differentiate asthma from COPD in some patients, as the symptoms and clinical features of both asthma and COPD overlap. These patients may be considered to have ACO. No formal definition or treatment algorithm exists for patients with coexistent asthma and COPD. The default therapy should be with ICSs, given their importance in asthma management,^[7]^ and additional LABA and/or LAMA are also needed in most cases.

### Difficult-to-treat v. severe asthma

In clinical practice, many patients may be on medication that would place them in the GINA step 5 category – traditionally part of the ‘severe asthma’ definition. Many patients may not be truly severe but rather difficult-to-treat, and attention to factors influencing asthma control as listed below may result in a reduction in medication requirements.

These subgroups of asthmatics should be evaluated at a specialist centre. It is estimated that between 5 and 10% of asthmatics are truly severe, and the differentiation from difficult-to-treat is important prior to escalating to expensive (biologic) and harmful (long-term oral corticosteroid) therapies. For those with potentially severe asthma characterised by ‘ongoing/uncontrolled symptoms despite optimised high-dose ICS-LABA combination therapy or requiring high-dose ICS-LABA to prevent it being uncontrolled’,^[1]^ a specific workup should be conducted to confirm the need for high-dose corticosteroids or biologic therapy.

The American Thoracic Society/European Respiratory Society (ATS/ERS) and GINA guidelines on severe asthma provide detailed guidance.^[50,51]^ Once the diagnosis of asthma is confirmed, work-up would include evaluation of:

- adherence and inhaler technique
- uncontrolled rhinosinusitis/(adults), nasal polyps
- psychological factors, e.g. personality trait, symptom perception, anxiety, depression
- smoking/smoking-related disease
- vocal cord dysfunction
- obesity and obstructive sleep apnoea
- hyperventilation syndrome
- hormonal influences, e.g. premenstrual, menarche, menopause, thyroid disorders
- medications, e.g. aspirin, non-steroidal anti-inflammatory drugs (NSAIDs), β-adrenergic blockers, angiotensin-converting enzyme inhibitors.
